# Identification of new *Saccharomyces cerevisiae* variants of the *MET2* and *SKP2* genes controlling the sulfur assimilation pathway and the production of undesirable sulfur compounds during alcoholic fermentation

**DOI:** 10.1186/s12934-015-0245-1

**Published:** 2015-05-08

**Authors:** Jessica Noble, Isabelle Sanchez, Bruno Blondin

**Affiliations:** Lallemand SAS, Blagnac, 31700 France; Institut Coopératif du Vin, Lattes, 34970 France; INRA, UMR1083 Sciences pour l’Oenologie, Montpellier, 34060 France; Supagro, UMR1083 Sciences pour l’Oenologie, Montpellier, 34060 France; UM1, UMR1083 Sciences pour l’Oenologie, Montpellier, 34060 France

**Keywords:** Sulfite, Sulfide, QTL, MET2, SKP2, Wine yeast

## Abstract

**Background:**

Wine yeasts can produce undesirable sulfur compounds during alcoholic fermentation, such as SO_2_ and H_2_S, in variable amounts depending mostly on the yeast strain but also on the conditions. However, although sulfur metabolism has been widely studied, some of the genetic determinants of differences in sulfite and/or sulfide production between wine yeast strains remain to be identified. In this study, we used an integrated approach to decipher the genetic determinants of variation in the production of undesirable sulfur compounds.

**Results:**

We examined the kinetics of SO_2_ production by two parental strains, one high and one low sulfite producer. These strains displayed similar production profiles but only the high-sulfite producer strain continued to produce SO_2_ in the stationary phase. Transcriptomic analysis revealed that the low-sulfite producer strain overexpressed genes of the sulfur assimilation pathway, which is the mark of a lower flux through the pathway consistent with a lower intracellular concentration in cysteine. A QTL mapping strategy then enabled us to identify *MET2* and *SKP2* as the genes responsible for these phenotypic differences between strains and we identified new variants of these genes in the low-sulfite producer strain. *MET2* influences the availability of a metabolic intermediate, O-acetylhomoserine, whereas *SKP2* affects the activity of a key enzyme of the sulfur assimilation branch of the pathway, the APS kinase, encoded by *MET14*. Furthermore, these genes also affected the production of propanol and acetaldehyde. These pleiotropic effects are probably linked to the influence of these genes on interconnected pathways and to the chemical reactivity of sulfite with other metabolites.

**Conclusions:**

This study provides new insight into the regulation of sulfur metabolism in wine yeasts and identifies variants of *MET2* and *SKP2* genes, that control the activity of both branches of the sulfur amino acid synthesis pathway and modulate sulfite/sulfide production and other related phenotypes. These results provide novel targets for the improvement of wine yeast strains.

**Electronic supplementary material:**

The online version of this article (doi:10.1186/s12934-015-0245-1) contains supplementary material, which is available to authorized users.

## Background

The control of metabolite production by yeast during alcoholic fermentation is a key issue for various fermented beverages, especially for wines. Among the metabolites released by yeast, those derived from sulfur metabolism are particularly important because they strongly influence the organoleptic quality of fermented beverages. Sulfites (SO_2_) and sulfide (H_2_S) are important metabolites in yeast metabolism and enology. They are key intermediates of the sulfur assimilation pathway and are also excreted by yeast into media. The excessive production of H_2_S can lead to off-flavors [1,2] and a high concentration of sulfites can delay the onset of malolactic fermentation by inhibiting lactic acid bacteria [3,4] and is also a source of health concerns. Indeed, given their toxicity, the final concentration of sulfites in wine is regulated by law. Therefore, the production of these compounds by yeast has to be tightly controlled at all steps of the fermentation process. The production of sulfites and sulfide by wine yeasts are highly strain-dependent, and despite strong selective processes some commercial yeast still produce high amounts of these sulfur compounds in some circumstances. The genetic basis of this variation between strains is unclear, although it has been proposed that sulfite reductase plays an important role in the production of H_2_S [5,6]. The identification of genes involved in such variations between strains will enable the optimization of the fermentation process and the construction of strains that produce low amounts of negative sulfur metabolites through breeding strategies.

Sulfur metabolism in wine yeasts has been widely studied and the pathways involved in sulfate assimilation and in the synthesis of sulfur-containing amino acids are well known [7]. The entire pathway is highly regulated and coordinated by several control mechanisms in response to the intracellular concentration of cysteine. These mechanisms notably involve the transcription of genes of the sulfur assimilation pathway, which are regulated by the binding of the transcription factor *MET4* to their promoter and its association with auxiliary factors, Met28p, Cbf1p, Met31p and Met32p [8-11]. *MET4* is controlled through an inhibitory mechanism mediated by *MET30* [12], which encodes an F-box protein that is part of an ubiquitin-proteasome complex [13,14]. This complex targets Met4p for degradation by the proteasome depending on the intracellular concentration of cysteine [15]. Furthermore, Natarjan *et al.* [16] showed that several genes of sulfur metabolism are also controlled by *GCN4*, which regulates the transcriptional activity of genes involved in amino acid synthesis either directly (*MET16* and *MET25*) or indirectly (*SUL1*, *SUL2*, *MET3*, *MET14*, *MET10*, *MET1*, *MET25*, *MET6*, *MET2*, *MET28* and *MET4*). In addition, Yoshida *et al.* [17] identified a new mechanism involving the F-box protein skp2p, which forms part of a complex, SCF^*SKP2*^, which controls the stability of Met14p, and regulates the transcription of the *STR1*, *2* and *4* genes.

The production of sulfites and sulfide depends on environmental factors including the concentration of nutrients in the media, and in particular that of nitrogen-containing compounds (ammonium, amino acids and especially sulfur-containing amino acids). Nitrogen concentration affects differently the production of SO_2_ and H_2_S: SO_2_ production is favored in the presence of high nitrogen concentrations [18], whereas H_2_S production is favored in nitrogen-deficient musts [19-21]. Supplementation with amino acids and/or ammonium can significantly affect SO_2_ and H_2_S production depending on the amount of added compound and the time of addition [19,20,22]. SO_2_ and H_2_S production is also affected by the concentration of sulfates and vitamins, such as pantothenate, and by pH and probably several other factors [23-26]. However, the largest source of variation in the production of sulfur compounds is the yeast strain itself. Wine yeasts produce sulfites at concentrations ranging from less than 10 mg/L to more than 100 mg/L [24]. Similarly, sulfide production is undetectable for some strains whereas other strains produce high amounts of sulfide [27,28]. Several genes involved in sulfur metabolism have been implicated in the ability of strains to produce sulfite and/or sulfide, suggesting that this phenotypic property is controlled by multiple genetic loci. Several studies have examined the effect of the deletion or the overexpression of genes of the sulfur assimilation pathway [29-32]. Some studies have also focused on variants of genes of the sulfur assimilation pathway that affect hydrogen sulfide formation, and in particular on variants of sulfite reductase, to identify mutants showing defects in the conversion of sulfite into sulfide [5,33,34]. However, the molecular basis responsible for differences in the production of sulfur compounds, and in particular that of sulfite, between yeast strains is still not fully understood. In this study, we used a QTL mapping strategy to search for genes responsible for phenotypic variation in SO_2_ and H_2_S production between yeast strains. This genetic approach is now widely used to study continuous phenotypes and has been successfully applied to several wine yeast traits, including complex traits governed by several loci [35-38]. We focused on two wine yeast strains; a high sulfite-producing strain and a low sulfite-producing strain. We built and characterized a population of recombined meiotic segregants to perform linkage analysis. This analysis revealed a double QTL on chromosome XIV containing two genes involved in sulfur metabolism, *MET2* and *SKP2*, which displayed allelic variations between the two strains. We show that these alleles modulate the production of sulfite, sulfide and acetaldehyde and we provide a new comprehensive view of the mechanisms responsible for variation in the production of sulfur compounds by wine yeasts.

## Results

### Characterization of sulfite production during alcoholic fermentation

**Figure 1:**
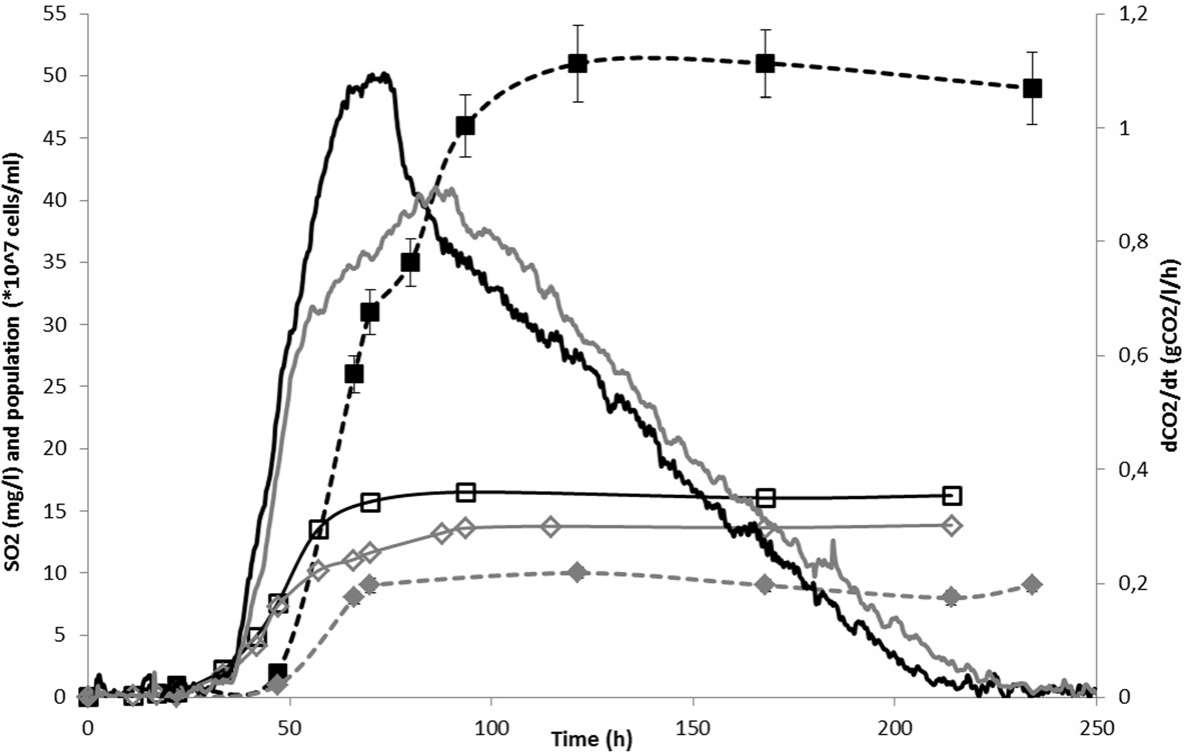
Kinetics of growth (open symbols), rate of CO_2_ (continuous lines) and SO_2_ (dotted lines and filled symbols) production for the parental strains JN10 (black lines and squares) and JN17 (gray lines and diamonds) in a synthetic must under conditions optimized for SO_2_ production. Parameters of fermentation: 425 mg/L assimilable nitrogen, 200 g/l equimolar mix of glucose and fructose, 16°C. SO_2_ concentrations and cell numbers are the mean of two replicates.

We selected two *Saccharomyces cerevisiae* strains, both of which were homozygous diploid derivatives of wine yeasts, which were previously shown to differ in their ability to produce sulfite: JN10, a high sulfite-producing strain, and JN17, a low sulfite-producing strain. We characterized the sulfite production of these two strains in a synthetic must under conditions that favor sulfite production: a high nitrogen content (425 mg/L) and a low temperature (16°C) as we determined in a preliminary study that a low temperature increased the SO2 production while the underlying mechanisms are still unknown. We monitored fermentation kinetics, including the rate of CO_2_ production, cell growth and sulfite concentration (Figure 1). These variables differed between the two strains. After a similar lag phase, the maximum rate of CO_2_ production was higher for the JN10 strain than for the JN17 strain, although the JN17 strain maintained a slightly higher rate of CO_2_ release during the beginning of the stationary phase. At the end of the fermentation, cells of the JN10 strain slightly outnumbered those of the JN17 strain (1.62 +/− 0.02 and 1.42 +/− 0.015 x10^8^ cells/mL, respectively). SO_2_ production began in the middle of the growth phase for both strains, and reached a maximum at the end of the growth phase for the JN17 strain. However, in the JN10 strain, SO_2_ continued to be produced during the beginning of the stationary phase. The concentration of sulfites was then stable until the end of the fermentation. As expected, the JN10 strain produced substantially more sulfite than the JN17 strain (final concentration 51 mg/L versus 10 mg/L, respectively).

Thus, sulfite production was tightly associated with growth phase for the low sulfite-producing strain. However, the high producing strain, JN10, continued to produce sulfite during the beginning of the stationary phase (about 40 mg/L SO_2_ was produced during this phase). This observation, in addition to the high release of sulfites by this strain, is probably explained by an overflow of sulfites during growth and a lack of adjustment of the sulfur pathway in response to growth arrest, as occurs in the JN17 strain.

We also analyzed other compounds that are directly or indirectly linked to sulfite production (Table 1). H_2_S is a metabolic intermediate immediately downstream from sulfite in the sulfur assimilation pathway and acetaldehyde binds to sulfur dioxide via its carbonyl group, its production was shown in previous study to be modulated by SO_2_ concentration [39]. Production of acetaldehyde in response to SO_2_ concentration can be seen as mechanism of protection of the yeasts to face the toxicity of sulfites and strains more resistant to SO_2_ have been shown to be higher acetaldehyde producers [40]. Acetaldehyde was quantified at the same points and in the same conditions as SO_2_ production whereas H_2_S production was assessed during fermentation in a nitrogen-deficient must (MS100, see Material and Methods) at 28°C. The JN10 strain produced more H_2_S and acetaldehyde than the JN17 strain, which is not surprising given the metabolic link between H_2_S, acetaldehyde and sulfites. We subsequently selected two approaches to investigate the mechanisms responsible for the differences between the parental strains: a transcriptomic approach, involving comparative whole-genome expression analysis, and a genetic approach involving QTL mapping to identify genomic regions associated with phenotypic differences.

**Table 1:** **Production of SO**_**2**_ **, H**_**2**_ **S and acetaldehyde of by the parental strains**

	**SO**_**2**_ **(mg/l)**	**H**_**2**_ **S (color of the H**_**2**_ **S detection strip)**	**Acetaldehyde (mg/l)**
JN10	50,40±3,05	High (black)	29,00±1,41
JN17	9,00±1,22	Low (white)	9,25±0,35

Acetaldehyde was determined in conditions previously defined as optimal for SO_2_ production analysis. H_2_S production was determined in a synthetic nitrogen-deficient must, at 28°C.

Values are the mean of five biological replicates for SO_2_ production and two biological replicates for acetaldehyde production. H_2_S production was estimated visually once.

### Comparative transcriptomic analysis of high and low sulfite-producing strains

We analyzed the transcriptome of the two yeast strains during the sulfite production phase, just after entry into the stationary phase, at the same stage of fermentation (36 g of CO_2_ released for both strains). This time point is the most representative of differences in sulfite production between strains, because it corresponds to the point at which sulfite production stopped in the JN17 strain but carried on in the JN10 strain. Moreover, a time point during the stationary phase was preferable to one during the transition between phases, because substantial transcriptomic alterations take place upon entry into the stationary phase [41]. RNA was extracted from both cell populations, and was labeled and hybridized to microarrays as described in Material and Methods.

**Figure 2:**
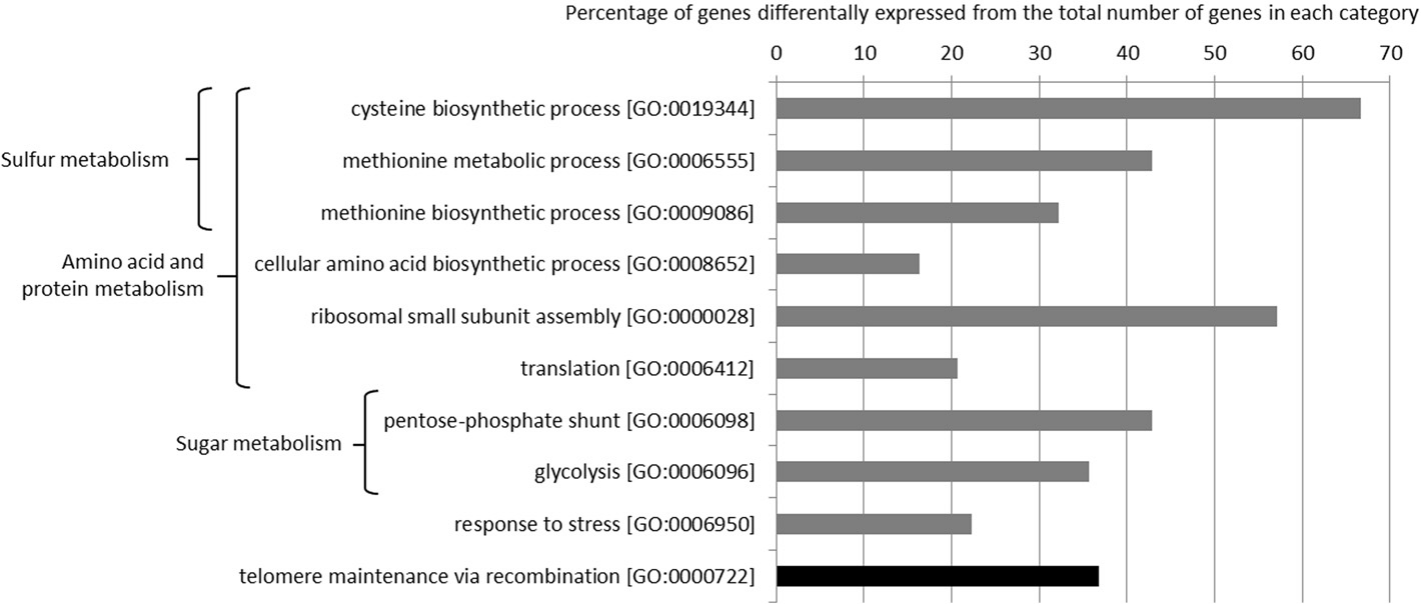
Classification of genes differentially expressed between the parental strains according to GO Biological Process categories. Bars show the percentage of affected genes from the total number of genes in each category. Gray bars show the categories of genes more strongly expressed in the JN17 strain and black bars show those more strongly expressed in the JN10 strain.

**Figure 3:**
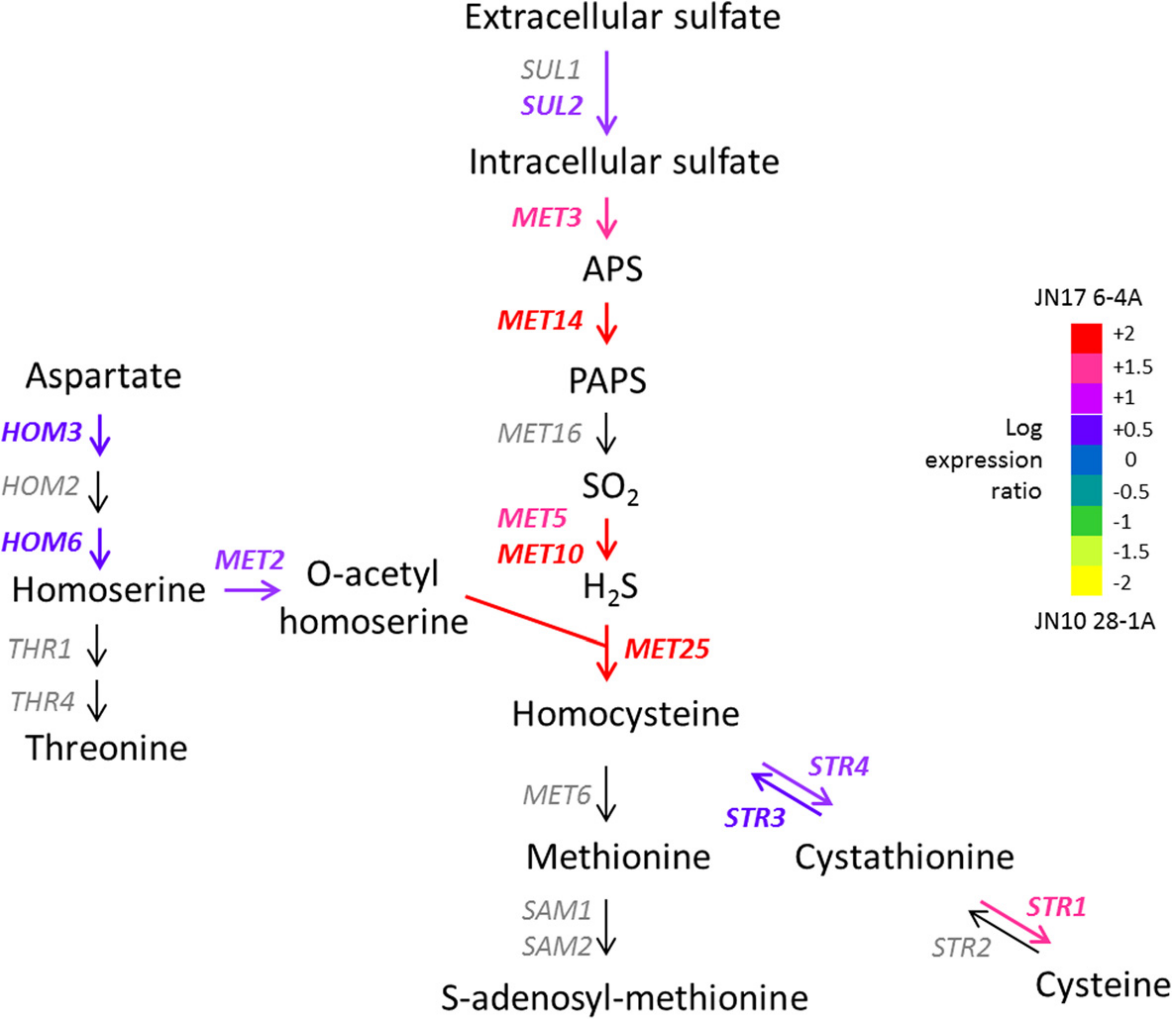
Schematic representation of the differential expression of the genes of the sulfur assimilation pathway between the parental strains. A color gradient represents the log of the expression ratio between the parental strains.

This analysis identified 627 differentially expressed genes at a 5% threshold, of which 274 were more strongly expressed in the JN10 strain than in the JN17 strain and 353 were more strongly expressed in the JN17 strain than in the JN10 strain (see Gene Expression Omnibus with the accession number GSE55083 for a complete dataset). The expression of 72 genes was at least two fold higher in the JN10 strain than in the JN17 strain whereas the expression of 111 genes was at least two fold higher in the JN17 strain than in the JN10 strain. We used gene ontology analysis to identify groups of genes or pathways among these differentially expressed genes, which revealed that genes involved in sulfur metabolism were differentially expressed between the two strains (Figures 2 and 3). The differential expression of a large number of genes is consistent with the coordinated regulation of all the genes of this pathway. Eight genes among 12 involved in cysteine biosynthesis were more strongly expressed in the low sulfite-producing strain, JN17, than in the high sulfite-producing strain, JN10. This result probably reflects a low intracellular concentration in cysteine as this pathway is regulated by feedback control [15]. The high expression of these genes is therefore consistent with the low flux of the sulfur pathway in the JN17 strain.

### Genetic study of phenotypic variation in SO_2_, H_2_S, and acetaldehyde production

We used a QTL mapping strategy to identify the molecular basis of phenotypic differences in SO_2_, H_2_S and acetaldehyde production. Stable haploid derivatives from the JN10 and JN17 strains (JN10 *ho:: KanMX* mat a and JN17 *ho:: KanMx* mat α) were crossed to obtain a hybrid, H53-A5. We then obtained 60 spores from 26 asci with an average viability of 2.3 spores per ascus (only one complete tetrad was obtained) and we analyzed the phenotypic and genotypic characteristics of this population of meiotic segregants.

**Figure 4:**
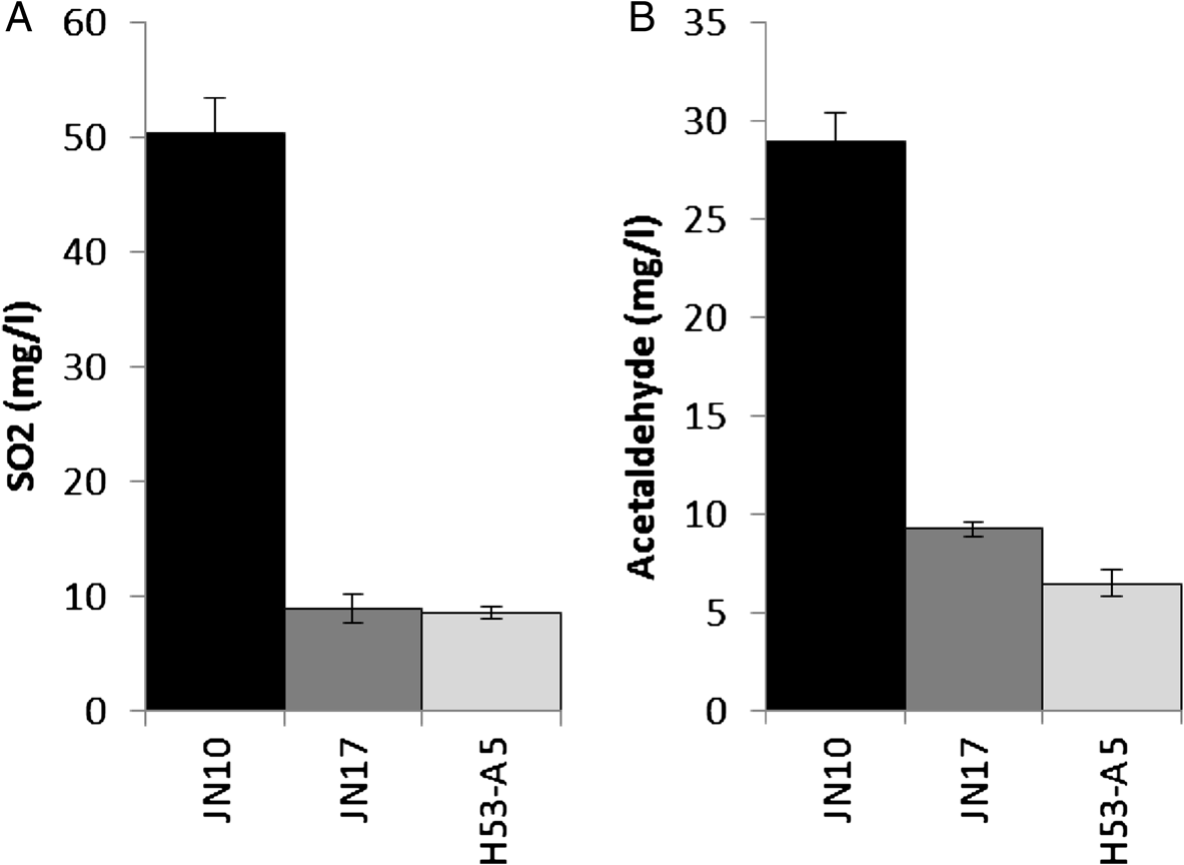
Production of SO_2_ **(A)** and acetaldehyde **(B)** by the parental strains (JN10 and JN17, respectively) and the hybrid (JN10/JN17). Values are the mean of five biological replicates for SO_2_ production by parental strains and three biological replicates for SO_2_ production by the hybrid. Two independent biological replicates were carried out to assess acetaldehyde production by the hybrid and parental strains.

**Figure 5:**
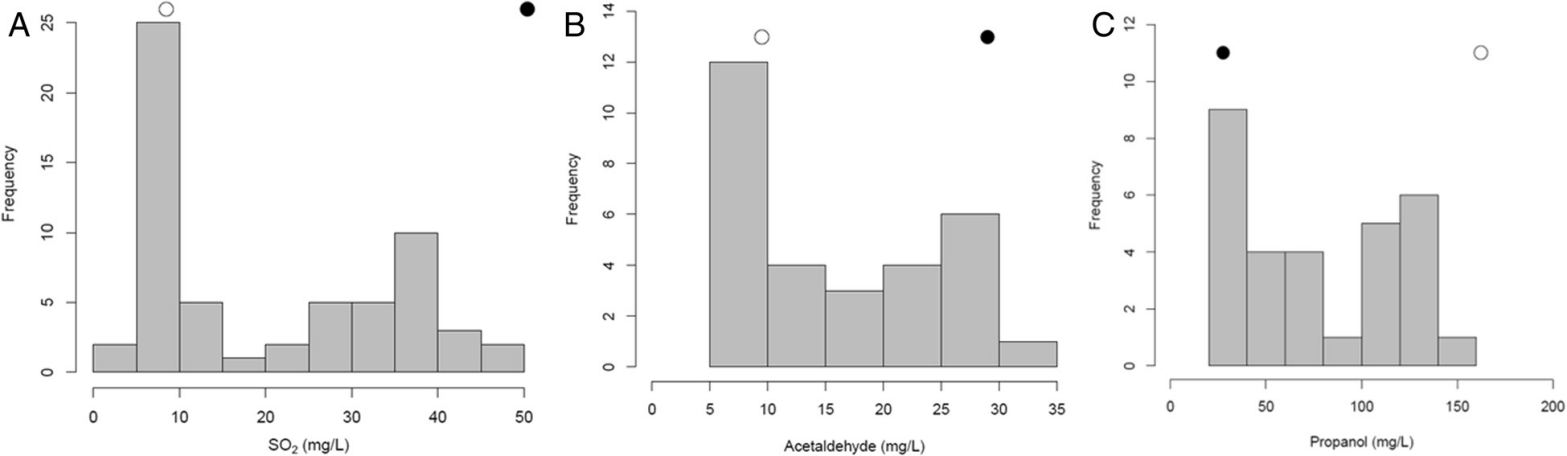
Distribution of phenotypes of SO_2_ production **(A)** among the segregant population, and distribution of phenotypes of acetaldehyde **(B)**, and propanol **(C)** in a subpopulation of 30 segregants randomly selected from the population. The parental strains are represented by circles above the diagram (JN10 black circle, JN17 white circle).

We assessed the production of sulfite, acetaldehyde and H_2_S as well as that of propanol and other volatile compounds in the hybrid and the meiotic segregants. The production of SO_2_, H_2_S and acetaldehyde was low in the hybrid and was similar or even lower than in the JN17 strain, which demonstrates the dominant character of the low sulfur metabolite-producing phenotype (Figure 4). The population of meiotic segregants displayed a bimodal distribution in terms of sulfite production and could be divided into a low SO_2_-producing group (<20 mg/L) and a high SO_2−_producing group (>20 mg/L). The population segregated equally into these groups suggesting that sulfite production is probably controlled by a major locus, which confers either low or high production phenotype (Figure 5A). Furthermore, the subgroup of high SO_2_-producing strains is continuously distributed, suggesting that several other loci modulate phenotype from moderate to high when the “high SO_2_ production” allele is present. In addition, few transgressive values were observed in the segregant population and the JN10 strain seemed to contain all the loci responsible for high sulfite production. We also determined the production of acetaldehyde and H_2_S in a subgroup of 30 randomly selected meiotic segregants. Acetaldehyde production tended to follow a bimodal distribution, similar to that of sulfite production, although the distinction between the two groups was less pronounced probably because of the smaller number of phenotyped segregants (Figure 5B). SO_2_ and acetaldehyde production were strongly correlated (Pearson’s correlation coefficient: 0.97), which is easily explained because acetaldehyde forms a complex with SO_2_. Indeed, the binding of acetaldehyde to SO_2_ probably increases proportionally as a function of SO_2_ production. Of the 30 meiotic segregants, half did not produce detectable levels of H_2_S whereas the other half produced H_2_S in varying amounts visually ranging from a slight blackening to a complete darkening of the strips. All the segregants producing no detectable H_2_S produced low amounts of SO_2_ whereas segregants producing detectable levels of H_2_S produced from less than 10 to more than 40 mg/L of SO_2_ (data not shown). Thus, the range of SO_2_ production by low SO_2_-producing strains is small and this phenotype is associated with undetectable levels of H_2_S, whereas H_2_S production by high SO_2_-producing strains shows substantial variation similar to the production of SO_2_ itself by these strains. These observations further reinforce the idea that “low SO_2_ production” is controlled by one major genetic determinant whereas the “high SO_2_ production” allele is modulated by several other loci. We also measured the production of propanol (Figure 5C) and other volatile compounds (data not shown) by gas chromatography. Propanol production by the subpopulation of 30 segregants showed a strong negative correlation with sulfite production (Pearson correlation coefficient −0.84) and also displayed a bimodal distribution. The production of sulfite may be related to that of propanol because these compounds share a common metabolic intermediate. Propanol is a derivative of α-ketobutyrate, which is derived from the degradation of threonine or from the interconversion of homocysteine to cysteine. Moreover, strong propanol production has been previously linked to the incapacity of some strains to produce H_2_S [42].

We then used comparative genome hybridization on high-density oligonucleotide microarrays (Affymetrix SG98) to identify molecular markers to distinguish the parental strains and we genotyped 28 randomly selected meiotic segregants from the population. These segregants originated from 16 different asci, containing one to four viable spores. From the genotyping data, we constructed a genetic map constituted of 1512 molecular markers, which were fairly uniformly distributed along the genome, although some regions were less covered than others, such as a region located in the middle of chromosome XVI (see Additional file 1 for a physical map of the molecular markers). The mean density of the markers was one marker every 8 kb. The genotype of the meiotic segregants was equally distributed between the two parental strains, with 49% of the markers from JN10 and 51% from JN17. We used the distribution of the markers among the 28 segregants to construct a recombination map (see Additional file 1), and we estimated that the mean number of recombination events was 212 per meiosis. This number is higher than that reported in the literature (approximately 80 events per meiosis) [43,44], possibly because the recombination capacity of our wine yeast strains is higher than that of other strains, and in particular, laboratory strains.

**Figure 6:**
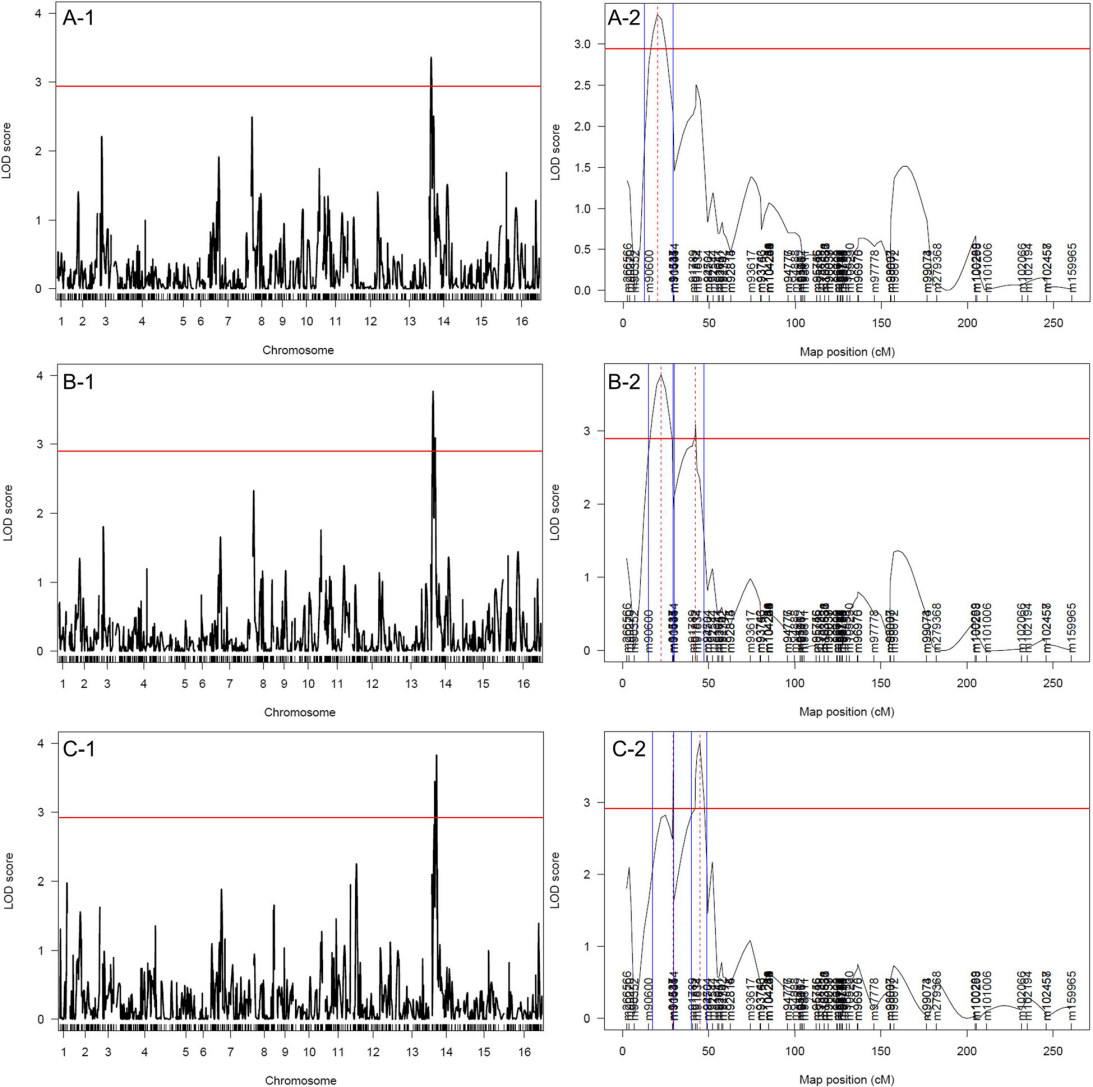
LOD score curves along the chromosomes for SO_2_ (**A**-1&2), acetaldehyde (**B**-1&2), and propanol (**C**-1&2) production phenotypes. A zoom on chromosome XIV is shown.

We used an interval mapping approach with a non-parametric model to perform linkage analysis. Two peaks of LOD score were observed on chromosome XIV for each phenotype (Figure 6 and Table 2). For the phenotypes of acetaldehyde and propanol production, both peaks were statistically significant whereas only one was above the significance threshold for the phenotype of SO_2_ production. The extremities of the regions varied slightly depending on the phenotype studied. Nonetheless, two non-overlapping QTL regions spanning from 37,204 kb to 86,919 kb and from 89,704 kb to 147,194 kb could be defined. Furthermore, a linkage analysis based on an extrapolation of the qualitative evaluation of H_2_S production into a binary phenotype (production/absence of production of H_2_S) revealed two peaks of QTL overlapping those regions and therefore reinforced the results (data not shown). We used the *Saccharomyces* genome database (www.yeastgenome.org) to explore the QTL regions and identify genes potentially associated with our phenotypes of interest. The first region was about 50 kb long and contained 35 ORF. Among them, we found a relevant candidate gene, *SKP2*, which encodes an F-box protein predicted to be part of an SCF ubiquitin protease complex that is involved in regulating the abundance of sulfur metabolism enzymes. The deletion of *SKP2* is associated with high H_2_S and SO_2_ production phenotype [17,45]. The second region was about 58 kb long and contained 38 ORF including a gene belonging to the sulfur assimilation pathway, *MET2. MET2* encodes the L-homoserine-O-acetyltransferase, which catalyzes the conversion of homoserine to O-acetyl homoserine, the first step of the methionine biosynthetic pathway. Inactivation of *MET2* promotes the accumulation of SO_2_ and H_2_S in brewer’s yeasts [30]. Given the known functions of these two genes, they were considered to be relevant candidates and were characterized further.

**Table 2:** **Location and characteristics of the QTL identified on the XIV chromosome**

**Phenotype**	**LOD score maximum**	**Significativity level of LOD score**	**Start position (pb)**	**End position (pb)**
SO2	3,36	2,94	37 204	86 919
Acetaldehyde	3,77	2,90	45 024	88 277
Acetaldehyde	3,09	2,90	89 704	142 204
Propanol	3,45	2,92	52 204	89 154
Propanol	3,83	2,92	119 704	147 194

### Sequencing and functional validation of the candidate genes

We sequenced both candidate genes and their promoter regions (from 370 and 384 pb upstream from the *SKP2* and *MET2* genes, respectively) in the two parental strains. Two SNPs were found in the *SKP2* coding sequence: one at position 1,048 pb and another at 1,070 pb. Both SNPs are non-synonymous and alter the amino acid sequence of Skp2. The SNP at 1,048 pb is a C > T transversion leading to the replacement of isoleucine with valine at position 350 (I350V) in the JN17 strain, and that at position 1,070 pb is a G > A transversion, leading to the replacement of threonine with isoleucine (T357I) in the JN17 strain. The I_357_ residue seems to be specific to the JN17 strain because the amino acid at position 350 is threonine in sequenced genomes of *S. cerevisiae* available in databases and in other species of the *Saccharomyces* genus. However, the V_350_ residue, which is present in JN17, seems to be more common than the I_350_ and is thus probably the ancestral allele. We also found a SNP in the *MET2* coding sequence at position 560 pb. This SNP leads to a C > G transversion resulting in the replacement of arginine with glycine at position 301 (R301G) in the JN17 strain. The *G*_*301*_ residue corresponds to that of the S288C reference strain sequence, whereas the *R*_*301*_ residue is present in several other *S. cerevisiae* strains and in other species of the *Saccharomyces* genus, and thus appears to be the ancestral allele.

**Figure 7:**
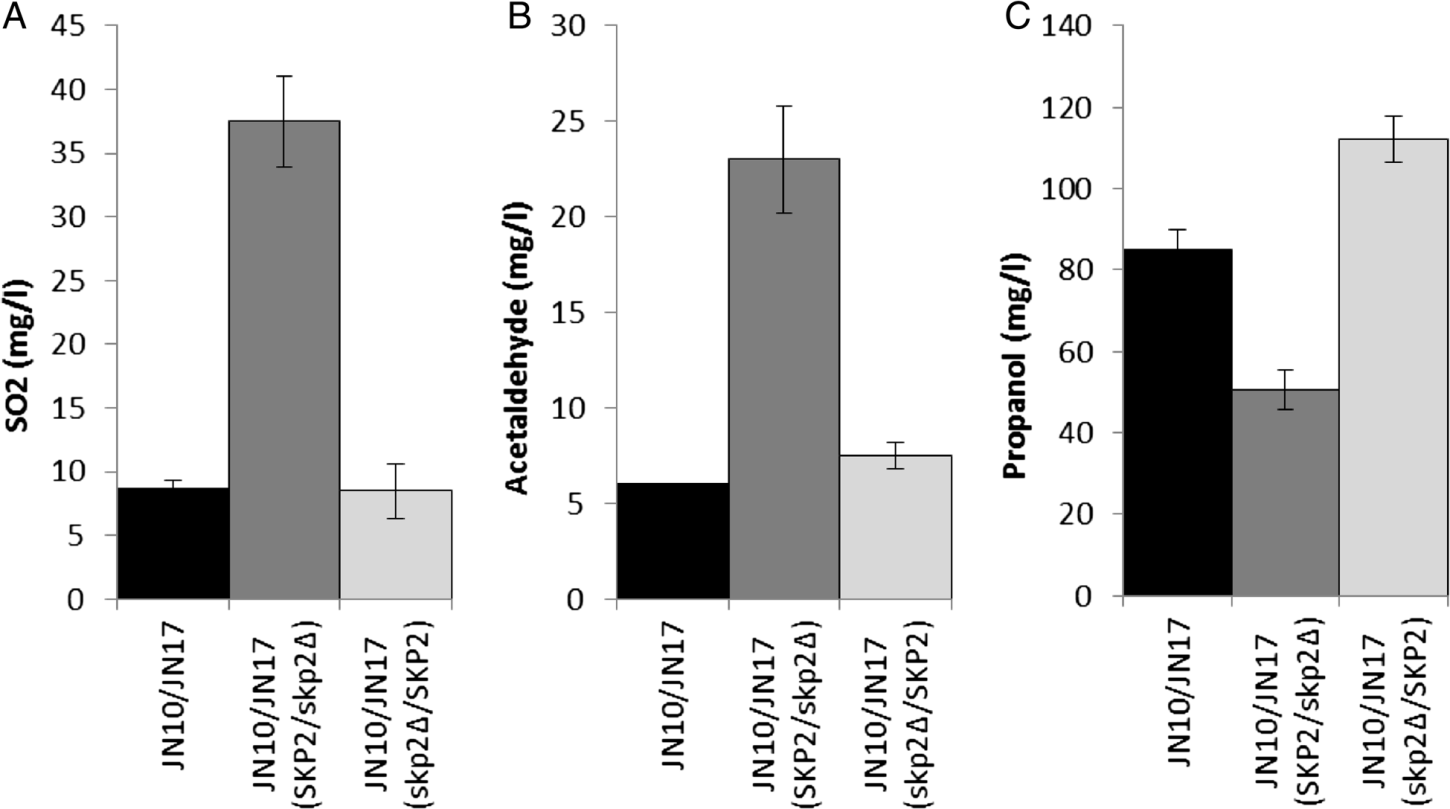
Production of SO_2_ **(A)**, acetaldehyde **(B)**, and propanol **(C)** by the hybrid H53-A5 (JN10/JN17) and the hemizygous strains (JN10/JN17 *SKP2*/*skp2*Δ and JN10/JN17 *skp2*Δ/*SKP2*, respectively). Values are the mean of five biological replicates for SO_2_ production by the parental strains, three biological replicates for SO_2_ production by the hybrid, two biological replicates for SO_2_ and acetaldehyde production by the hemizygous strains and two technical replicates for propanol production by all strains.

We carried out reciprocal hemizygosity analysis [46] of *SKP2* and allelic replacement of *MET2* to evaluate the effect of these genetic variants on phenotype and we examined SO_2_, H_2_S, acetaldehyde and propanol production in the resulting strains. Hemizygous diploids were constructed by crossing either the JN17 strain with a derivative of JN10 in which *SKP2* was disrupted or the JN10 strain with a derivative of JN17 bearing a disrupted *SKP2* gene. The hemizygous strain possessing the *SKP2*^JN10^ copy produced a high quantity of sulfite, similar to that of the parental strain JN10, whereas the hemizygous strain possessing only the *SKP2*^JN17^ allele produced a very low amount of sulfite, equivalent to that of the JN17 strain (Figure 7). Acetaldehyde production was correlated with that of sulfite, with a high production associated with the *SKP2* allele of JN10. Similarly, the strain possessing the *SKP2*^JN10^ copy produced a strong detectable level of H_2_S whereas the hemizygous strain possessing the *SKP2*^JN17^ copy did not produce any detectable H_2_S. Variations in propanol production between strains were more nuanced than for the other compounds. The hybrid produced quantities of propanol that were intermediate between the parental strains. The hemizygous strain possessing the *SKP2*^JN10^ copy produced lower quantities of propanol than the hybrid strain whereas the hemizygous strain possessing the *SKP2*^JN17^ allele produced higher quantities than the hybrid strain. Overall, these results show that the *SKP2* allele strongly influences the production of sulfur metabolites, acetaldehyde and propanol.

**Figure 8:**
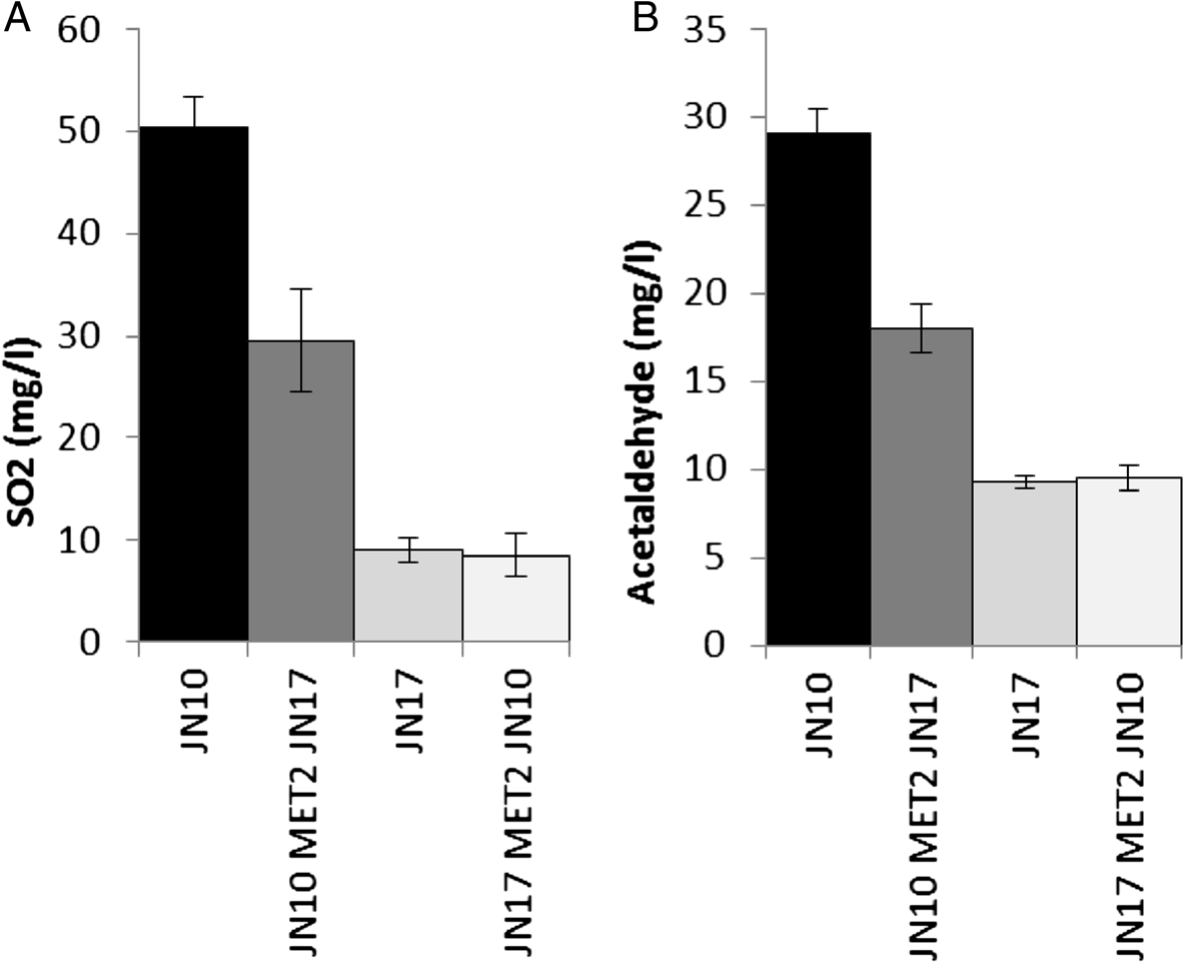
Production of SO_2_ **(A)** and acetaldehyde **(B)** by the parental strains(JN10 and JN17, respectively) and the strains in which the *MET2* gene of one parental strain was replaced with the *MET2* allele of the other by allelic replacement(JN10 *MET2* ^JN17^ and JN17 *MET2* ^JN10^, respectively). Values are the mean of five biological replicates for SO_2_ production by the parental strains, three biological replicates for SO_2_ production by the hybrid, and two biological replicates for SO_2_ and acetaldehyde production by the strains in which the *MET2* gene of one parental strain was replaced with the *MET2* allele of the other.

Sulfite production was substantially lower in the JN10 strain possessing the *MET2*^JN17^ allele than in the parental J10 strain (Figure 8). However, the reciprocal replacement of the *MET2* gene with the *MET2*^JN10^ allele in the JN17 background did not affect sulfite production. The phenotype of acetaldehyde production showed a similar trend to that of sulfite. H_2_S production was high in the J10 parental strain, undetectable in the J17 parental strain, and intermediate in both strains resulting from allelic replacement. The presence of the *MET2*^JN17^ allele in the JN10 genetic background impaired SO_2_, H_2_S and acetaldehyde production. However, the presence of the *MET2*^JN10^ allele in the JN17 background seemed to be counterbalanced by other loci, probably *SKP2*, because only the H_2_S production was affected by allelic replacement.

## Discussion

We report here a physiological and genetic study of two wine yeast strains that differ substantially in their ability to produce sulfite. This analysis identifies new variants of the *MET2* and *SKP2* genes that influence the production of sulfite, sulfide, acetaldehyde and propanol under conditions of alcoholic fermentation.

The physiological analysis of these strains revealed large differences in their ability to produce SO_2_. Indeed, the parental JN10 strain produced at least five times more sulfite than the parental JN17 strain, and we also identified large differences between strains in the production of metabolites directly related to sulfites, including acetaldehyde and sulfide. Kinetic analysis of the fermentation process revealed that the high production of sulfite was related to a prolonged production phase, which persisted after the end of the growth phase whereas for the JN17 strain showed a tight relation between growth phase and sulfite production. Indeed, about 80% of total sulfite produced by the JN10 strain occurred during the stationary phase. The coordination between growth phase and sulfite production was confirmed under other conditions of temperature or nitrogen content (data not shown) and is consistent with previous findings [29]. This coupling may be due to the activation of the sulfur pathway following the rapid depletion of sulfur-containing amino-acids as described by Rossignol *et al.* [41]. A transcriptomic analysis just after the entry in stationary phase then revealed that components of the sulfur metabolism pathway were more strongly expressed in the low sulfite-producing strain than in the high sulfite-producing strain. This reflects the low availability of sulfur-containing amino acids in the low sulfite-producing strain, because the expression of this pathway is controlled by feedback. Nevertheless, this strain did not seem to be affected by a deprivation in sulfur-containing amino acids, because its fermentation capacity and cell growth were closed to that of the high sulfite-producing strain. We then used a QTL mapping strategy to identify genetic variants associated with phenotypic differences between strains. Analysis of the distribution of phenotypes in a population of meiotic segregants provided insight into the genetic determinism of sulfite production. This analysis suggested that a major locus confers a low sulfite-producing phenotype and is probably also involved in the control of related phenotypes, such as acetaldehyde, sulfide and propanol production. Linkage analysis of the meiotic segregants identified a double QTL located on chromosome XIV containing two relevant genes related to sulfur metabolism, *SKP2* and *MET2*.

The *SKP2* gene was previously identified by Yoshida *et al.*, as involved in the control of the sulfur assimilation pathway [17]. Indeed, Met14p, the adenylylsulfate kinase, responsible for the conversion of 5′-adenylylsulfate (APS) to 3′-5′-adenylylsulfate (PAPS), is more stable in an *skp2*-null mutant than in a wild type background. This finding may be explained by the implication of *SKP2* in an SCF (Skp1 Cdc53 F-box protein) ubiquitin protease complex. The ubiquitin proteasome system regulates the abundance of many proteins involved in a wide variety of pathways. In this system, proteins are targeted for degradation by the binding of ubiquitin (Ub). Ub is first activated by a Ub-activating enzyme E1 and is then transferred to a Ub-conjugating enzyme, E2. Finally, the association of E2 with a ubiquitin ligase, E3, guides the transfer of Ub to the substrate. SCF complexes are a class of E3 ubiquitin ligases. The substrate specificity of SCF complexes is determined by the interchangeable F-box protein. SCF^MET30^ is a well-known SCF complex involved in the regulation of the sulfur assimilation pathway. A target of SCF^MET30^ is the transcription factor *MET4*, which regulates the expression of methionine biosynthetic genes [12,14]. Skp2p possesses an F-box domain, a degenerated motif of about 40 amino acids that enables its interaction with Skp1p [47]. Skp2p also interacts with Met14p [17] and thus Met14p is probably one of the targets of the SCF^SKP2^ complex. The amino acid sequence of Skp2 differs at two positions between the two parental strains. These substitutions were not located in the F-box domain and therefore should not alter the interaction with Skp1p. However, they may potentially affect the efficiency substrate recognition. In the case of the JN17 strain, we hypothesize that Skp2 recognizes Met14p with high efficiency, thus promoting its degradation. The low stability of Met14p potentially limits the flux through the assimilatory part of the pathway, thus impairing the conversion of sulfate to sulfites. This probably leads to a low rate of synthesis of SO_2_ and H_2_S. Consistent with this hypothesis, the overexpression of *MET14* promotes SO_2_ production and its conversion to H_2_S [29]. The coding sequence of the *MET2* gene differed at one position between the two parental strains. This substitution may affect the efficiency of homoserine trans-acetylase and probably leads to the synthesis of higher amounts of O-acetylhomoserine and a greater incorporation of H_2_S into carbon skeleton in the JN17 strain than in the JN10 strain.

The high production and release of sulfite into media by the JN10 strain may thus be explained by the following factors: (1) the high stability of Met14p leading to high flux through the reductive part of the sulfite assimilation pathway; and (2) the synthesis of inadequate amounts of O-acetylhomoserine, which is the precursor of the incorporation of H_2_S into homocysteine, resulting in a disequilibrium between the synthesis and incorporation of SO_2_/H_2_S.

*SKP2* appeared to influence strongly the flux through the sulfate assimilation pathway and the production of SO_2_ and acetaldehyde. Allelic replacement of the *MET2* gene and complementary experiments confirmed the strength of this influence. First, SO_2_ and acetaldehyde production were similar between a JN17 strain carrying the *MET2*^JN10^ allele and the parental JN17 strain. We then analyzed SO_2_ production by 30 meiotic segregants in a must supplemented with 1 g/L of threonine (see Additional file 2). Threonine concentration is involved in a feedback mechanism that controls the activity of aspartate kinase (which is encoded by *HOM3*) [48]. Aspartate kinase is responsible for the first step of the conversion of aspartate to homoserine, which is subsequently converted to O-acetylhomoserine. Thus, high threonine concentrations impair the activity of the branch of the pathway that produces carbon precursors needed for the incorporation of H_2_S. Segregants producing low amounts of SO_2_ were not affected by the addition of threonine; however, segregants producing moderate to high amounts of SO_2_ strongly responded to threonine and produced between 30 and 70% more SO_2_ than in conditions without added threonine. Genetic analysis of the segregants identified no relationship between the identity of the *MET2* allele and response to added threonine. Thus, the *SKP2*^JN17^ allele controls sulfite production regardless of the identity of the *MET2* allele, and limits sulfite production even under highly favorable conditions. We also examined SO_2_ production by parental strains lacking a functional *MET2* allele in media supplemented with methionine to restore their growth. Sulfite production was twice as high in the JN17Δ*MET2* strain than in the parental JN17 strain (40 +/− 3 and 20 +/− 2 mg/L, respectively) and was substantially higher in the JN10Δ*MET2* strain than in the parental JN10 strain (129 +/− 4 and 40 +/− 2 mg/L, respectively) (data not shown). This demonstrates that sulfite production, and indirectly that of the acetaldehyde, are predominantly controlled by *SKP2* and not *MET2*. This finding can also be applied to the branch of the sulfur assimilation pathway leading to the synthesis of O-acetylhomoserine.

Nonetheless, an efficient homoserine trans-acetylase is probably required for the complete control of H_2_S production. Indeed, *SKP2* tightly regulates the rate of conversion of sulfate into sulfite, but it is probably the rate of incorporation of H_2_S into carbon precursors also affects its release into the medium. Complementary experiments involving allelic replacement of both genes should provide a clear demonstration of this assumed additive effect.

The impact of the identified genes on other metabolites of importance in enology could also be of great interest. In our study, we could observe that there was no influence on the glycerol production, whereas a link through the redox balance could have been awaited. We noticed that the difference in glycerol production between the parental strains is weak (5.46 g/l +/− 0.01 and 5.30 g/l +/− 0.04 for the strains JN10 and JN17 respectively). A sample of 15 segregants revealed that the glycerol production varied from 4.87 mg/l +/− 0.01 to 6.31 mg/l +/− 0.09 but there was no relation between the glycerol and the SO_2_ production (Pearson coefficient of 0.2781). The variation range in the sulfur metabolites seemed to be too low to impact the glycerol production. On another hand, sulfur compounds derivative from H_2_S such as ethanethiol, methanethiol or methyl sulfide and disulfide could also be analyzed as we expect a decrease of those compounds responsible for off-flavors proportional to that of H_2_S. Such a diminution has already been observed on natural must when comparing the JN17 strain with other commercial wine strains for the methanethiol for instance (data not shown).

Moreover, other loci with minor effects may be involved in the modulation of the high production of sulfite. Additional minor QTLs could maybe be identified with an increased number of genotyped segregants and/or a higher number of molecular markers thus increasing the density of markers and filling the gaps in their distribution along the genetic map.

## Conclusions

The molecular basis of many of the enological properties of wine yeasts remains unknown. Although many studies have investigated sulfur metabolism in wine yeast, some of the genetic variants responsible for differences in sulfite and sulfide production between strains remain to be characterized. Emphasis in previous studies has often been placed on sulfide production, which is responsible for off-flavors, and sulfite reductase mutants that cannot convert sulfite into sulfide have been developed. However, these strains release large amounts of sulfites into media [5,6], which negatively affects the organoleptic properties of wine, delays malolactic fermentation, and has implications for human health. Therefore, much interest has been placed in methods to control sulfite production. Current trends in winemaking tend towards a diminution or even a total abolition of sulfite use and winemakers need to be provided with low sulfite-producing strains.

In this study, we used a QTL mapping strategy coupled with physiologic and transcriptomic studies to identify mechanisms underlying the control of sulfite production as well as phenotypes related to this process. We show that the *SKP2* and *MET2* genes influence SO_2_, H_2_S and acetaldehyde productions and we identified new variants of these genes in a low sulfite-producing strain. These variants control two aspects of sulfur metabolism: sulfate assimilation and the synthesis of carbon precursors. The rarity of these alleles, in particular the *SKP2*^JN17^ allele, suggests that alternative mechanisms and genes/alleles combination can also restrict sulfite and sulfide production in other low producers yeast strains. Nevertheless the robust low sulfite-producing phenotype associated with the combination of these alleles suggests that their transfer to any high producer strain of wine yeast should be sufficient to control sulfite/sulfide and acetaldehyde production in most cases. The transfer of these alleles via a non-GMO route may be possible through backcrossing approaches that have been previously used to improve wine yeasts [49]. Furthermore, these genes are genetically linked, and would therefore be easy to transfer simultaneously during backcrossing cycles. Our study thus provides new perspectives for the improvement of wine yeast. The transfer of these alleles to commercial strains can be considered an alternative to common strategies currently used to control H_2_S production such as those involving the restriction of sulfite reductase activity which result in the uncontrolled release of SO_2_ [5].

## Material and methods

### Yeast strains

The two parental yeast strains, JN10 and JN17, were obtained by dissecting asci of two *Saccharomyces cerevisiae* wine yeasts, isolated from grapes and used commercially. Those strains are available in our collection under the reference codes MTF1832 and MTF1833 and are accessible upon request. Like most wine yeast strains, these yeasts were homothallic heterozygous diploids and thus gave rise to monosporic diploids. These derivatives were assumed to be completely homozygous because they underwent self-diploidization. They were selected according to their phenotypic similarity with their corresponding parental strain. Stable haploids were then obtained through the disruption of the *HO* gene using short flanking homologous sequences to facilitate further breeding. A KanMX4 cassette conferring resistance to geneticin (G418) was amplified from a plasmid (pUG6) with 60-mer primers that contained a stretch of 40 nucleotides identical to the upstream or downstream sequence of the *HO* gene flanked by 20 nucleotides homologous to the plasmid, as follows: pHOdelF: 5′- ATGCTTTCTG AAAACACGAC TATTCTGATG GCTAACGGTG CTTCGTACGC TGCAGGTC -3′ and pHOdelR: 5′- TTAGCAGATG CGCGCACCTG CGTTGTTACC ACAACTCTT TAGTGGATCT GATATCACCT A -3′. The strains were transformed according to the procedure described by Schiestl and Gietz [50]. The integration of the cassette in transformants was verified by PCR on genomic DNA with a primer located upstream (pHOdelverifF TGTTGAAGCATGATGAAGCG) or downstream (pHOdelverifR TGAAACAAATCAGTGCCGGT) from the insertion and primers in the KanMX gene (pkanP1r 5′-GCTAAATGTACGGGCGAC-3′ and pkanP2f 5′-TCGCCTCGACATCATCTG-3′). Transformation usually affects only one copy of the gene in diploid strains; therefore, transformants were induced to sporulate and stable haploid spores disrupted for the *HO* gene were selected. The mating type of the haploids was determined through crossing experiments with reference strains of known mating type. The strains JN10 *ho:: KanMX4* mat a and JN17 *ho:: KanMX4* mat α were crossed to obtain a hybrid, H53-A5. This hybrid was induced to sporulate and asci were dissected to generate a collection of 60 meiotic segregants. A list of strains used in this study is presented in Table 3.

**Table 3:** **List of** ***Saccharomyces cerevisiae*** **strains used in this study**

**Name**	**Origin**	**Genotype**
JN10	Homozygous diploid obtained from a high sulfite producer wine yeast	Homozygous diploid HO/HO
JN17	Homozygous diploid obtained from a low sulfite producer wine yeast	Homozygous diploid HO/HO
JN10 mat a	Haploid spore of JN10	Haploid ho:: KanMX mat a
JN17 mat α	Haploid spore of JN17	Haploid ho:: KanMX mat α
H53-A5	Hybrid of JN10 mat a and JN17 mat α	Diploid
JN10 *MET2* JN17	Allelic replacement for the *MET2* gene in a JN10 background	Haploid *MET2* JN17
JN17 *MET2* JN10	Allelic replacement for the *MET2* gene in a JN17 background	Haploid *MET2* JN10
JN10/JN17 (*SKP2*/*skp2*Δ)	Hemizygote between JN10 and JN17 *skp2*:: HPH	Diploid *SKP2* JN10/*skp2* JN17:: HPH
JN10/JN17 (*skp2*Δ/*SKP2*)	Hemizygote between JN10 *skp2*:: HPH and JN17	Diploid *skp2* JN10:: HPH/*SKP2* JN17

### Growth and fermentation media

Yeast were grown in YEPD medium (2% glucose, 1% yeast extract, 2% bactopeptone, and 2% agar if necessary) at 28°C. Geneticin (G418, 200 μg/mL) was added to solid YEPD medium to select for transformed yeast strains. Sporulation was induced by transferring yeast cells onto sporulation medium (1% potassium acetate, 0.1% yeast extract, 0.05% glucose, 0.002% adenine and 2% agar) after 48 h of growth at 28°C on presporulation medium (10% glucose, 1% yeast extract, 0.5% bactopeptone and 2% agar). Plates of sporulation medium were incubated at 28°C for at least four days. Microdissection of asci was performed with a micromanipulator (Singer Instruments) on a micromanipulation medium (0.2% yeast extract, 0.2% glucose, 2% ultrapure agar).

Fermentation was carried out in synthetic musts mimicking natural must, as described by Bely *et al.* [51] with some minor modifications. Sugar was provided by an equimolar mix of glucose and fructose at a combined total of 200 g/L and the content of anaerobic factors was 75% lower than that described by Bely *et al.* Assimilable nitrogen content varied from 100 mg/L to 425 mg/L. Temperature was maintained either at 16°C or at 28°C depending on the phenotype measured. Fermentation media was inoculated at 10^6^ cells/mL after two sequential pre-cultures. A first pre-culture was performed in liquid YEPD for one day, and was transferred to synthetic must in agitated flasks that were left to grow for another day. Fermentation units of 1.2 L and 0.3 L were used. Both kinds of bioreactors were equipped with airlocks to maintain anaerobiosis and were under permanent stirring.

### Determination of phenotypic variables

CO_2_ release was monitored by weight loss, which was assessed either automatically for the 1.2 L unit (one acquisition every 20 minutes) or manually for the 0.3 L unit. The rate of CO_2_ production was calculated with a method of polynomial smoothing from the weight loss data of the 1.2 L fermentation units. Cell number was determined with an electronic particle counter (Coulter, Beckman). Sulfite production was determined in small fermentation units at 16°C on a nitrogen rich media after 90% of the fermentation process was complete. The media contained 425 mg/L of assimilable nitrogen because these conditions were determined to be optimal for the assessment of differences in sulfite production between strains (data not shown). SO_2_ concentration was measured with an enzymatic UV assay (r-Biopharm) according to the manufacturer’s instructions. This method measures the total amount of sulfite (free and carbonyl-bound sulfite) with a detection limit of 0.3 mg/l.

H_2_S detection strips (Fluka) were used to determine H_2_S production and were placed in the CO_2_ release flow in the fermenter bells of 1.2 L fermentation units. H_2_S production was assessed at 28°C in nitrogen-poor media (100 mg/l assimilable nitrogen), because these conditions have been previously described to favor H_2_S production. The amount of H_2_S was estimated visually according to the blackening of the strips and provides a binary response, production or lack of production of H_2_S, with a detection limit about 0.5 to 1 μg/l [52,53]. Acetaldehyde production was determined with an enzymatic UV method in the same conditions described for SO_2_ production. Fifty microliters of supernatant were mixed with 1500 μL of a premix solution (1 mg/mL NAD in a buffered solution of pyrophosphate acid, pH9). The formation of NADH after the oxidation of acetaldehyde by aldehyde dehydrogenase (10 μL of an enzymatic suspension at 45 U/mL, Sigma) was determined from optical density at 340 nm.

Propanol concentrations were measured by head-space gas chromatography (GC Agilent 6890) in the same conditions used to determine SO_2_ production.

### Analysis of gene expression

Gene expression was analyzed with microarrays spotted with the 6308 oligonucleotides (70 mer) of the *S. cerevisiae* Oligoset (Operon) in duplicate on UltraGap chips (Biochip Platform, Toulouse, France). RNA was extracted with Trizol reagent with a method adapted from Chomczynski and Sacchi [54]. Reverse transcription and labeling were performed with a ChipShot direct labeling and clean-up system kit (Promega) according to the manufacturer’s instructions. Microarray hybridization was carried out with a Pronto Universal Microarray kit (Corning) according to the manufacturer’s instructions. Two biological replicates were included according to a *dye swap* design. Microarrays were scanned with a GenePix pro 3 scanner (Axon Instruments). Data were processed with the R software (R2.9.2) and the Limma package [55-59]. Intra-array normalization was carried out with the print-tip loess method and inter-array normalization with the quantile method. Differentially expressed genes were identified through a linear model approach and a Benjamini-Hochberg method was used to adjust the *p*-values [60]. A gene was considered as differentially expressed at a significance level of 5% if its adjusted *p*-value was less than 0.05. The complete data set is available at Gene Expression Omnibus with the accession number GSE55083. Statistical analysis to determine functional groups of genes that were over-represented in the data set was performed with the web-based tool FunSpec [61] (available online at http://funspec.med.utoronto.ca/, *p*-value <0.05, and Bonferroni correction) and genes were classified with the GO database.

### Genotyping with high-density oligonucleotide microarrays

Genomic DNA was extracted with the Genomic Tip 100G kit (Qiagen) according to the manufacturer’s instructions. Three independent extractions were performed for each parental strain and one for each meiotic segregant. Genomic DNA was fragmented, labeled and hybridized onto Yeast Genome S98 arrays (Affymetrix) by the genomic platform ProfilExpert (IFR Neuroscience, Lyon). Microarrays were scanned by an Affymetrix scanner. Raw data were submitted to multiple filters and statistical analyses with the R software as previously described [43] to identify informative markers. Molecular markers were positioned on a physical map and on a genetic map, using a conversion factor of 3000 bp for 1 cM.

### QTL mapping

Linkage analysis between phenotypic and genotypic datasets was performed by an interval mapping method [62] implemented in the R/qtl package [63]. A non-parametric model was applied to all the studied phenotypes, except for H_2_S production, which was analyzed with a binary model because of its semi-quantitative character [64]. The values 1 and 2 of the arbitrary color scale were combined into one single value. The significance level was determined through permutation tests (1000 permutations). The confidence interval for the location of each QTL was defined as 1-LOD support interval: the region in which the LOD score is within 1 unit of the linkage peak.

### Gene sequencing

The *SKP2* and the *MET2* genes were amplified with the following primers: pSKP2F4, 5′-TCATCATGTTACCGTGGAACA-3′ and pSKP2R, 5′-AGTCCACTACAAAAAGTCAT-3′ for *SKP2*; pMET2F, 5′-TTGTTAGTGGCTCCCCAC-3′ and pMET2R, 5′-ATGTTATGCCTGAGGTAT-3′ for *MET2*. Both genes were sequenced by Eurofins MWG (Ebersberg, Germany) with the above-listed amplification primers and the following primers for fragments between 500 and 800 pb: pMET2seqW1, 5′-TAACGACTTAGCATTCGA-3′; pMET2seqW2, 5′-CACCGCATCTTCTTCGGA-3′; pMET2seqC1, 5′-TGTGGATTTGTAGGGAGT-3′; pMET2seqC2, 5′-TCACCAGCTTCATTCAGT-3′ and pSKP2seq1, 5′-CTACAATTTGATTACGAATG-3′, pSKP2seq2, 5′-CAGTAAATTCGACTTATTGT-3′, pSKP2seq3, 5′-TGGGAACATCTAGCAAGAAC-3′.

### Construction of strains for functional analysis

Allelic replacement was performed to confirm functionally the implication of *MET2* in phenotypic variation. The *MET2* gene was deleted in one haploid parental strain and replaced with a cassette conferring resistance to ClonNat (plasmid pAG25, NAT1 gene) by the transformation protocol described previously in the “Yeast strains” section. The following primers were used for the deletion: pMET2delF 5′-GACATCAGCAAGACATTCTGCCTGGTGCATATCGTGGTCTTGCCTCGTCCCCGCCGGGTC-3′ and pMET2delR 5′-CAGCCAAAAATTCTTGTTCTGAATATGTGAACAGTCCATCCAGTATAGCGACCAGCATTC-3′, and the replacement was verified with pClonNatF 5′-CTCACATCACATCCGAACAT-3′ and pMET2R primers. Strains lacking a functional *MET2* gene were auxotroph for methionine. Transformants were then transformed again with a plasmid encoding the *MET2* gene of the other parental strain that was amplified previously. The *MET2* gene was amplified with the primers pMET2F4 5′-AAGAATATGGTTGCTCTGGC-3′ and pMET2R2 5′-TGCGACTTCGGTATGTGCT-3′. Transformants that were prototroph for methionine were selected and the following strains were obtained: JN10 *ho:: KanMX4* mat a *MET2*^JN17^ and JN17 *ho:: KanMX4* mat α *MET2*^JN10^.

A counter-selection approach could not be used to test the implication of *SKP2* in phenotypic variation because the inactivation of this gene does not lead to a discriminant phenotype. Therefore, we performed a reciprocal hemizygosity analysis and deleted the *SKP2* gene in one haploid parental strain through replacement with a cassette conferring resistance to ClonNat (plasmid pAG25, NAT1 gene). The following primers were used for the deletion: pSKP2delF 5′-AAGTTGAACCGCATTTTCAAACGTTCAAACCAACCGAATCTGCCTCGTCCCCGCCGGGTC-3′ and pSKP2delR 5′-TGCATAAATATGCTATATAAAGTCCACTACAAAAAGTCATCAGTATAGCGACCAGCATTC-3′ and the replacement was verified with pClonNatF and pSKP2R primers. The strain deleted for *SKP2* was then crossed with the other parental strain resulting in a diploid strain possessing only one active copy of the *SKP2* gene, thus giving rise to the strains JN10/JN17 Δ*skp2 and* JN17/JN10 Δ*skp2*.

## Additional files

Additional file 1:
**Physical map of the molecular markers.** The x-axis shows the genomic location of markers (expressed in nucleotides) and each line of the y-axis shows one chromosome. Additional file 2:
**Production of SO**_**2**_ **by the parental strains and a subgroup of 28 meiotic segregants in nitrogen rich media at 16°C with or without added threonine (1 g/L).** Segregants carrying the JN10 *MET2* allele are shown with black bars and those carrying the JN17 allele are shown with gray bars.

## References

[1] Swiegers JH, Pretorius IS (2007). Modulation of volatile sulfur compound by wine yeast. Appl Microbiol Biotechnol.

[2] Mendes-Ferreira A, Barbosa C, Falco V, Leao C, Mendes-Faia A (2009). The production of hydrogen sulfide and other aroma compounds by wine yeasts of *Saccharomyces cerevisiae* in synthetic media with different nitrogen concentrations. J Ind Microbiol Biotechnol.

[3] Henick-Kling T, Park YH (1994). Considerations for the use of yeasts and bacteria starter cultures: SO2 and timing of inoculation. Am J Enol Vitic.

[4] Carreté R, Vidal MT, Bordons A, Constanti M (2002). Inhibitory effect of sulfur dioxide and other stress compounds in wine on the ATPase activity of *Oenococcus oeni*. FEMS Microbiol Lett.

[5] Cordente AG, Heinrich A, Pretorius IS, Swiegers JH (2009). Isolation of sulfite reductase variants of a commercial wine yeast with significantly reduced hydrogen sulfide production. FEMS Yeast Res.

[6] Linderholm A, Dietzel K, Hirst M, Bisson LF (2010). Identification of MET10-932 and characterization as an allele reducing hydrogen sulfide formation in wine strains of *saccharomyces cerevisiae*. Appl Environ Microbiol.

[7] Thomas D, Surdin-Kerjan Y (1997). Metabolism of sulfur amino acids in Saccharomyces cerevisiae. Mol Biol Rev.

[8] Blaiseau PL, Thomas D (1998). Multiple transcriptional activation complexes tether the yeast activator Met4 to DNA. EMBO J.

[9] Blaiseau PL, Isnard AD, Surdin-Kerjan Y, Thomas D (1997). Met31p and Met32p, two related zinc finger proteins, are involved in transcriptional regulation of yeast sulfur amino acid metabolism. Mol Cell Biol.

[10] Kuras L, Cherest H, Surdin-Kerjan Y, Thomas D (1996). A heteromeric complex containing the centromere binding factor 1 and two basic leucine zipper factors, Met4 and Met28, mediates the transcription activation of yeast sulfur metabolism. EMBO J.

[11] Thomas D, Jacquemin I, Surdin-Kerjan Y (1992). MET4, a leucine zipper protein, and centromere-binding factor 1 are both required for transcriptional activation of sulfur metabolism in Saccharomyces cerevisiae. Mol Cell Biol.

[12] Thomas D, Kuras L, Barbey R, Cherest H, Blaiseau P, Surdin-Kerjan Y (1995). Met30p, a yeast transcriptional inhibitor that responds to S- adenosylmethionine, is an essential protein with WD40 repeats. Mol Cell Biol.

[13] Craig K, Tyers M (1999). The F-box: a new motif for ubiquitin dependent proteolysis in cell cycle regulation and signal transduction. Prog Biophys Mol Biol.

[14] Rouillon A, Barbey R, Patton EE, Tyers M, Thomas D (2000). Feedback-regulated degradation of the transcriptional activator Met4 is triggered by the SCFMet30 complex. EMBO J.

[15] Hansen J, Johannesen PF (2000). Cysteine is essential for transcriptional regulation of the sulfur assimilation genes in *Saccharomyces cerevisiae*. Mol Gen Genet.

[16] Natarajan K, Meyer MR, Jackson BM, Slade D, Roberts C, Hinnebusch AG (2001). Transcriptional profiling shows that Gcn4p is a master regulator of gene expression during amino acid starvation in yeast. Mol Cell Biol.

[17] Yoshida S, Imoto J, Minato T, Oouchi R, Kamada Y, Tomita M (2011). A novel mechanism regulates H_2_S and SO_2_ production in *Saccharomyces cerevisiae*. Yeast.

[18] Duan W, Roddick F, Higgins V, Rogers P (2004). A parallel analysis of H_2_S and SO_2_ formation by brewing yeast in response to sulfur-containing amino acids and ammonium ions. J Am Soc Brew Chem.

[19] Giudici P, Kunkee RE (1994). The effect of nitrogen deficiency and sulfur-containing amino acids on the reduction of sulfate to hydrogen sulfide by wine yeasts. Am J Enol Vitic.

[20] Jiranek V, Langridge P, Henschke PA (1995). Regulation of hydrogen sulfide liberation in wine-producing *Saccharomyces cerevisiae* strains by assimilable nitrogen. Appl Environ Microbiol.

[21] Vos PJA, Gray RS (1979). The origin and control of hydrogen sulfide during fermentation of grape must. Am J Enol Vitic.

[22] Ugliano M, Fedrizzi B, Siebert T, Travis B, Magno F, Versini G (2009). Effect of nitrogen supplementation and saccharomyces species on hydrogen sulfide and other volatile sulfur compounds in shiraz fermentation and wine. J Agric Food Chem.

[23] Eschenbruch R (1974). Sulfite and sulfide formation during winemaking – a review. Am J Enol Vitic.

[24] Eschenbruch R, Bonish P (1976). Production of sulphite and sulphide by low-and high-sulphite forming wine yeasts. Arch Microbiol.

[25] Eschenbruch R, Bonish P (1976). The influence of pH on sulphite formation by yeasts. Arch Microbiol.

[26] Wainwright T (1970). Hydrogen sulphide production by yeast under conditions of methionine, pantothenate or vitamin B6 deficiency. J Gen Microbiol.

[27] Kumar GR, Ramakrishnan V, Bisson LF (2010). Survey of hydrogen sulfide production in wine strains of *saccharomyces cerevisiae*. Am J Enol Vitic.

[28] Spiropoulos A, Tanaka J, Flerianos I, Bisson LF (2000). Characterization of hydrogen sulfide formation in commercial and natural wine isolates of *Saccharomyces*. Am J Enol Vitic.

[29] Donalies UE, Stahl U (2002). Increasing sulphite formation in *Saccharomyces cerevisiae* by overexpression of *MET14* and *SSU1*. Yeast.

[30] Hansen J, Kielland-brandt MC (1996). Inactivation of *MET2* in brewer’s yeast increases the level of sulfite in beer. J Biotechnol.

[31] Hansen J, Kielland-Brandt MC (1996). Inactivation of *MET10* in brewer’s yeast specifically increases SO_2_ formation during beer production. Nat Biotech.

[32] Spiropoulos A, Bisson LF (2000). *MET17* and hydrogen sulfide formation in *Saccharomyces cerevisiae*. Appl Environ Microbiol.

[33] Linderholm AL, Olineka TL, Hong Y, Bisson LF (2006). Allele diversity among genes of the sulfate reduction pathway in wine strains of *Saccharomyces cerevisiae*. Am J Enol Vitic.

[34] Linderholm AL, Findleton CL, Kumar G, Hong Y, Bisson LF (2008). Identification of genes affecting hydrogen sulfide formation in *Saccharomyces cerevisiae*. Appl Environ Microbiol.

[35] Ambroset C, Petit M, Brion C, Sanchez I, Delobel P, Guérin C (2011). Deciphering the molecular basis of wine yeast fermentation traits using a combined genetic and genomic approach. G3.

[36] Marullo P, Aigle M, Bely M, Masneuf-Pomarede I, Durrens P, Dubourdieu D (2007). Single QTL mapping and nucleotide-level resolution of a physiologic trait in wine *Saccharomyces cerevisiae* strains. FEMS Yeast Res.

[37] Steyer D, Ambroset C, Brion C, Claudel P, Delobel P, Sanchez I (2012). QTL mapping of the production of wine aroma compounds by yeast. BMC Genomics.

[38] Swinnen S, Schaerlaekens K, Pais T, Claesen J, Hubmann G, Yang Y (2012). Identification of novel causative genes determining the complex trait of high ethanol tolerance in yeast using pooled-segregant whole-genome sequence analysis. Genome Res.

[39] Rankine CC, Pocock KF (1969). Influence of yeast strain on binding of sulphur dioxide in wines, and on its formation during fermentation. J Sci Fd Agric.

[40] Casalone E, Colella CM, Daly S, Gallori E, Moriani L (1992). Mechanism of resistance to sulfite in *Saccharomyces cerevisiae*. Curr Genet.

[41] Rossignol T, Dulau L, Julien A, Blondin B (2003). Genome-wide monitoring of wine yeast gene expression during alcoholic fermentation. Yeast.

[42] Guidici P, Zambonelli C, Kunkee RE (1993). Increased production of n-propanol in wine by yeast strains having an impaired ability to form hydrogen sulfide. Am J Enol Vitic.

[43] Brem RB, Yvert G, Clinton R, Kruglyak L (2002). Genetic dissection of transcriptional regulation in budding yeast. Science.

[44] Cubillos FA, Billi E, Zorgo E, Parts L, Fargier P, Omholt S (2011). Assessing the complex architecture of polygenic traits in diverged yeast populations. Mol Ecol.

[45] Yoshida S, Imoto J, Minato T, Oouchi R, Sugihara M, Imai T (2008). Development of bottom-fermenting *saccharomyces* strains that produce high SO_2_ levels, using integrated metabolome and transcriptome analysis. Appl Environ Microbiol.

[46] Steinmetz LM, Sinha H, Richards D, Spiegelman JI, Oefner PJ, McCusker JH (2002). Dissecting the architecture of a quantitative trait locus in yeast. Nature.

[47] Bai C, Sen P, Hofmann K, Ma L, Goebl M, Harper JW (1996). *SKP1* connects cell cycle regulators to the ubiquitin proteolysis machinery through a novel motif, the F-Box. Cell.

[48] Stadtman ER, Cohen GN, Lebras G, Robichon-Szulmajster H (1961). Feedback inhibition and repression of aspartokinase activity in *Escherichia coli* and *Saccharomyces cerevisiae*. J Biol Chem.

[49] Marullo P, Mansour C, Dufour M, Albertin W, Sicard D, Bely M (2009). Genetic improvement of thermo-tolerance in wine *Saccharomyces cerevisiae* strains by a backcross approach. FEMS Yeast Res.

[50] Schiestl RH, Gietz RD (1989). High efficiency transformation of intact yeast cells using single stranded nucleic acids as a carrier. Curr Genet.

[51] Bely M, Sablayrolles JM, Barre P (1990). Description of alcoholic fermentation kinetics: its variability and significance. Am J Enol Vitic.

[52] Park SK (2008). Development of a method to measure hydrogen sulfide in wine fermentation. J Microbiol Biotechnol.

[53] Ugliano M, Henschke PA (2010). Comparison of three methods for accurate quantification of hydrogen sulfide during fermentation. Analytical Chimica Acta.

[54] Chomczynski P, Sacchi N (1987). Single-step method of RNA isolation by acid guanidinium thiocyanate-phenol-chloroform extraction. Anal Biochem.

[55] R Development Core Team: R: a language and environment for statistical computing. R Fundation for Statistical Computing, Vienna, Austria. 2011 URL http://www.R-project.org/.

[56] Smyth GK (2004). Linear models and empirical bayes methods for assessing differential expression in microarray experiments. Stat Appl Genet Mol Biol.

[57] Smyth GK, Gentleman R, Carey V, Dudoit S, Irizarry R, Huber W (2005). Limma: linear models for microarray data. Bioinformatics and computational biology solutions using R and bioconductor.

[58] Smyth GK, Speed T (2003). Normalization of cDNA microarray data. Methods.

[59] Smyth GK, Michaud J, Scott HS (2005). Use of within-array replicate spots for assessing differential expression in microarray experiments. Bioinformatics.

[60] Benjamini Y, Hochberg Y (1995). Controlling the false discovery rate: a practical and powerful approach to multiple testing. J R Stat Soc Ser B Methodol.

[61] Robinson MD, Grigull J, Mohammad N, Hughes TR (2002). FunSpec: a web-based cluster interpreter for yeast. BMC Bioinformatics.

[62] Lander ES, Botstein D (1989). Mapping mendelian factors underlying quantitative traits using RFLP linkage maps. Genetics.

[63] Broman KW, Wu H, Sen Ś, Churchill GA (2003). R/qtl: QTL mapping in experimental crosses. Bioinformatics.

[64] Broman KW, Sen S. A guide to QTL mapping with R/qtl. Statistics for Biology and Health 2009 ISBN978-0-387-92124-2

