# Supplementary figures and images for: Identification of new Saccharomyces cerevisiae variants of the MET2 and SKP2 genes controlling the sulfur assimilation pathway and the production of undesirable sulfur compounds during alcoholic fermentation
Source: Microb Cell Fact. 2015 May 8;14:68. doi: 10.1186/s12934-015-0245-1 (PMC4432976; doi:10.1186/s12934-015-0245-1)

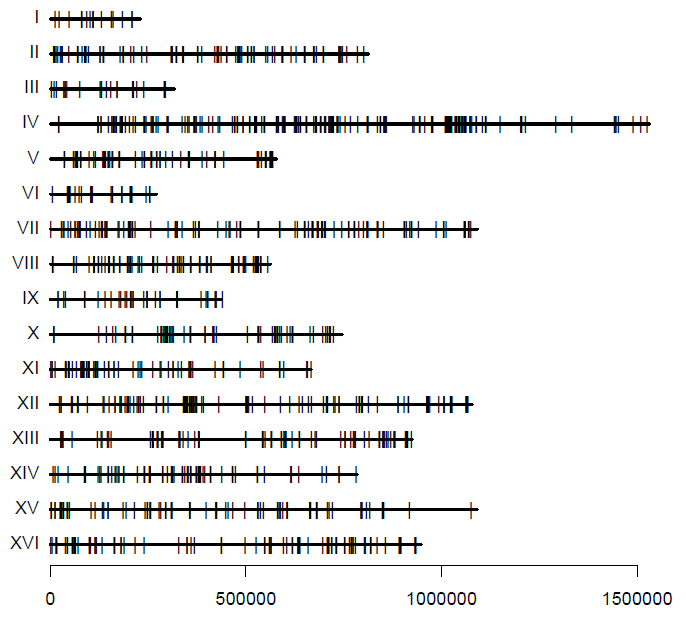

Supplement: Additional file 1: — Physical map of the molecular markers. The x-axis shows the genomic location of markers (expressed in nucleotides) and each line of the y-axis shows one chromosome. [file 12934_2015_245_MOESM1_ESM.png]

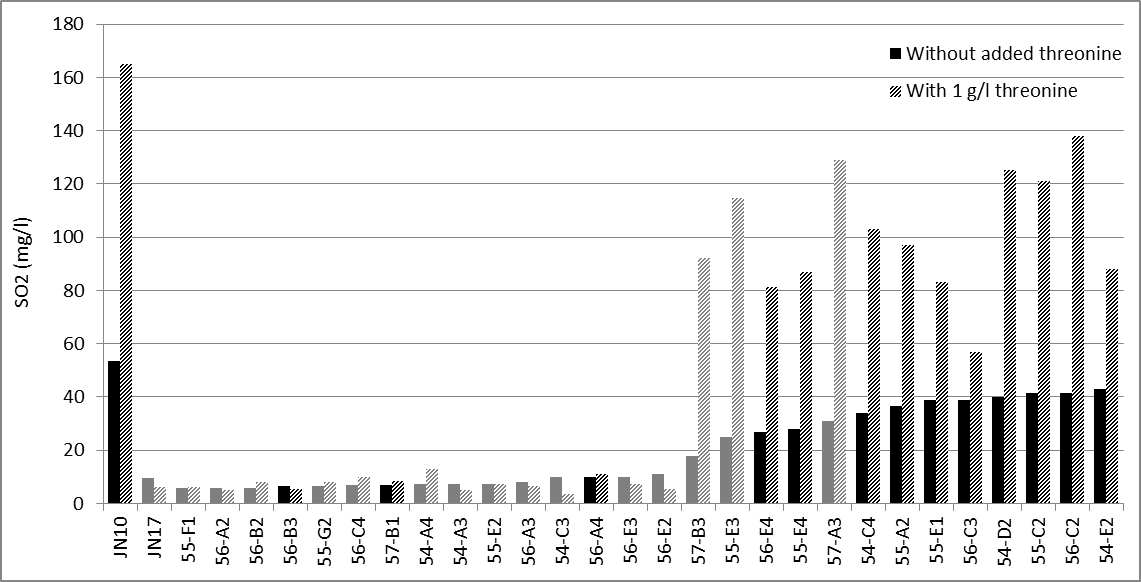

Supplement: Additional file 2: — Production of SO 2 by the parental strains and a subgroup of 28 meiotic segregants in nitrogen rich media at 16°C with or without added threonine (1 g/L). Segregants carrying the JN10 MET2 allele are shown with black bars and those carrying the JN17 allele are shown with gray bars. [file 12934_2015_245_MOESM2_ESM.png]
